# Are Specific Components of Executive Function Associated with the Functions of Non-Suicidal Self-Injury? A Network Analysis of Chinese University Students with Past-Year NSSI

**DOI:** 10.3390/bs16071156

**Published:** 2026-07-09

**Authors:** Bo Tian, Huili Ma, Yang He, Dong Wang, Minghao Man

**Affiliations:** 1 Department of Neurosurgery, Tangdu Hospital, The Fourth Military Medical University, Xi’an 710038, China; 2 School of Psychology, Shanghai Normal University, Shanghai 200234, China; mhl2692@163.com (H.M.);; 3 Department of Geriatric Psychiatry, Suzhou Mental Health Center, Suzhou Guangji Hospital, The Affiliated Guangji Hospital of Soochow University, Suzhou 415100, China

**Keywords:** non-suicidal self-injury, executive function, self-injury functions, network analysis, university students

## Abstract

Non-suicidal self-injury (NSSI) is a significant public health concern among university students. Accumulating evidence suggests that deficits in executive function (EF) are associated with NSSI; however, little is known about how specific EF components relate to the specific psychological functions that motivate self-injury. This study aimed to investigate the fine-grained associations between EF dimensions and NSSI functions in university students with past-year NSSI using network analysis. Altogether, 1078 Chinese university students (82.6% female; Mage = 19.07, SD = 1.03) who had engaged in at least one NSSI behavior in the past year were enrolled. Executive function was assessed using the Adolescent Executive Function Scale, which measures three dimensions: inhibitory control, cognitive flexibility, and working memory. NSSI functions were assessed using the 19-item function subscale of the Adolescent Non-Suicidal Self-Injury Assessment Questionnaire. A Gaussian graphical model was used to estimate the network. Expected influence (EI) and bridge expected influence (BEI) were computed to identify core nodes and bridge nodes, respectively. Of the 231 possible edges, 117 (50.65%) were non-zero, with the strongest within-community edge linking “coping with sadness and disappointment” (F3) and “expressing despair and hopelessness” (F4). Across communities, the most prominent edge was between “inhibitory control” and “having a desire to harm myself and cannot stop”. The three highest EI values were observed for “having a desire to harm myself and cannot stop,” “letting others make changes,” and “self-punishment”. “Inhibitory control” showed the highest BEI in the EF community, while “having a desire to harm myself and cannot stop” showed the highest BEI in the NSSI function community. Both EI and BEI demonstrated excellent stability (CS coefficients = 0.75). In university students with past-year NSSI, inhibitory control and the uncontrollable urge to self-injure function as critical bridge nodes linking executive dysfunction to NSSI functions, while self-punishment and interpersonal influence motives emerge as central drivers in the network. These findings highlight inhibitory control, the uncontrollable urge to self-injure, self-punishment, and interpersonal influence as promising targets for precision interventions aimed at disrupting the maladaptive cycle maintaining NSSI in this population.

## 1. Introduction

Non-suicidal self-injury (NSSI) refers to the direct and deliberate destruction of one’s own body tissue without suicidal intent, encompassing behaviors such as cutting, scratching, and hitting, which are not socially sanctioned and are clearly distinct from suicide attempts (Klonsky & Muehlenkamp, 2007; Nock, 2010). The transition to university represents a critical developmental period during which students face multiple stressors, including identity transitions, academic pressure, and new social relationships, making them a high-risk population for NSSI (Kroshus et al., 2021; Boyne & Hamza, 2022). Indeed, the age range of 20 to 24 years has been identified as the second peak period for NSSI after adolescence (Gandhi et al., 2018). Prevalence estimates range from 10.3% in Europe to 17.8% in Brazil among college students (Kiekens et al., 2019; da Silva Bandeira et al., 2022), while in China, rates range from 11.0% to 35.0% (Lu et al., 2020). NSSI not only causes physical harm but also significantly elevates the risk of subsequent suicide attempts (Whitlock et al., 2013), and has emerged as a major public health concern worldwide. However, pain and injury are inherently aversive experiences that organisms are biologically programmed to avoid. The compelling motives that lead individuals to override this self-preservation instinct and engage in deliberate self-harm warrant systematic investigation.

NSSI functions refer to the motives or reinforcers that drive the behavior (Klonsky, 2007). A four-function model classifies these into automatic negative reinforcement (ANR), automatic positive reinforcement (APR), social negative reinforcement (SNR), and social positive reinforcement (SPR) (Nock & Prinstein, 2004; Nock, 2009). A meta-analysis found that intrapersonal functions (66% to 81%) are endorsed substantially more than social functions (33% to 56%), with affect regulation being the most common (Taylor et al., 2018; Klonsky, 2009). Critically, individuals rarely report a single function; most endorse multiple functions simultaneously (Coppersmith et al., 2021), and greater functional diversity is associated with more severe psychopathology and elevated suicide risk (Klonsky & Olino, 2008; Turner et al., 2013). Understanding NSSI functions thus holds direct clinical utility for tailoring interventions (Klonsky, 2007; Nock, 2009).

While emotional factors have been a primary focus in NSSI research, the onset and maintenance of NSSI are not driven solely by affective processes; cognitive processes also play a critical role (Hasking et al., 2017). Executive function (EF), a set of higher-order cognitive processes that regulate thought, emotion, and behavior toward goal-directed outcomes, is particularly important in this context (Diamond, 2013). EF is typically conceptualized as comprising three core dimensions: inhibitory control, cognitive flexibility, and working memory (Miyake et al., 2000). Inhibitory control refers to the ability to suppress impulsive responses, and deficits in this dimension have been consistently linked to self-injurious behavior (McHugh et al., 2019; Fikke et al., 2011). Cognitive flexibility, which involves shifting between mental sets to adapt to changing demands, has also been implicated in NSSI, with deficits in this domain associated with more frequent self-injury (C. Wang et al., 2025). Working memory supports the maintenance and manipulation of information, and impairments in this domain have been identified in individuals who self-injure (Hu et al., 2021; Fikke et al., 2011). However, most prior studies have relied on aggregate measures or group comparisons, focusing primarily on NSSI occurrence or frequency rather than on the specific functions the behavior serves. Consequently, the fine-grained associations between specific EF components and specific NSSI functions remain largely unexplored.

Network analysis provides a powerful framework to address this gap. Network theory conceptualizes mental disorders as arising from dynamic interactions among symptoms and related factors, which are represented as nodes, with their associations represented as edges (Borsboom, 2017; Borsboom & Cramer, 2013). This approach enables researchers to investigate potential pathological pathways at the dimension level and to understand how distinct symptom communities interconnect (Fried & Cramer, 2017; P. J. Jones et al., 2021). Furthermore, network analysis provides centrality indices, such as expected influence, which quantifies the overall importance of a node within the network (Robinaugh et al., 2016), and bridge expected influence, which identifies bridge nodes that connect different communities (P. J. Jones et al., 2021). Central nodes play a key role in activating other nodes and maintaining the overall network structure (Borsboom, 2017; Cai et al., 2022), while bridge nodes facilitate the spread of activation from one community to another and are critical for understanding how distinct constructs co-occur (P. J. Jones et al., 2021). For example, a network analysis of executive function and disinhibited eating in youth identified general inhibitory control and objective palatable food intake as key bridge nodes linking the two communities (Byrne et al., 2021). Recent network studies on NSSI have further demonstrated the utility of this approach: intrapersonal functions such as affect regulation have been found to be directly connected to persistent NSSI and suicide risk (Szewczuk-Bogusławska et al., 2024), and NSSI functional nodes related to coping with distress have been shown to be closely linked to depressive symptoms in adolescents (Guan et al., 2024). However, to our knowledge, no network analytic study has yet systematically examined how EF dimensions interconnect with NSSI functions in young adults with NSSI.

Therefore, the present study constructed a joint network of EF dimensions and NSSI functions in a sample of Chinese university students who had engaged in NSSI over the past year. The aims were: (1) to examine the fine-grained connections between specific EF components and specific NSSI functions; (2) to identify core nodes that play key activating roles in the overall network; and (3) to pinpoint key bridge nodes linking the EF and NSSI function communities, thereby providing potential targets for precision interventions.

## 2. Methods

### 2.1. Participants

This study used an online survey hosted on the Wenjuanxing platform (https://www.wjx.cn/ (accessed on 31 December 2025)). Data were collected between 31 December 2025 and 20 January 2026. University students were recruited through convenience sampling from several universities across four provincial-level administrative divisions in China (Zhejiang, Shaanxi, and Hunan Provinces, and Shanghai Municipality). Participants were eligible for inclusion if they (1) were aged 18–22 years; (2) had engaged in at least one non-suicidal self-injury (NSSI) behavior in the past year; (3) had no self-reported history of severe mental disorders (e.g., schizophrenia, bipolar disorder); and (4) were able to complete the online questionnaire independently. Electronic informed consent was obtained after participants were informed about the purpose and nature of the research, as well as their right to withdraw at any time. The anonymity of the study was emphasized to encourage honest responses. To control data quality, participants who failed two embedded attention-check questions (e.g., “Please select ‘4’ for this item”) were excluded from the analysis. A total of 1078 valid responses were retained. The study was approved by the relevant institutional ethics committee and complied with the Declaration of Helsinki.

### 2.2. Measures

#### 2.2.1. NSSI and Its Functions

NSSI history was assessed using the 12-item NSSI-behavior (NSSI-B) subscale of the Adolescent Non-Suicidal Self-Injury Assessment Questionnaire (ANSAQ; Wan et al., 2018). Items capture the frequency of self-injurious methods over the past year (e.g., “intentionally pinching myself”) on a 5-point scale from 0 (never) to 4 (always). Consistent with the DSM-5 definition of self-harm frequency and prior research (Yuan et al., 2023), participants who endorsed at least one behavior (score ≥ 1 on any item) were classified as having NSSI. All 1078 participants met this criterion.

NSSI functions were measured with the 19-item function subscale (NSSI-F) of the ANSAQ. Items evaluate the reasons for engaging in self-injury (e.g., “to make myself feel less lonely”) on a 5-point scale from 0 (completely disagree) to 4 (completely agree), with higher scores indicating stronger endorsement of that function. In the present study, Cronbach’s α = 0.905.

#### 2.2.2. Executive Function (EF)

EF was assessed using the Adolescent Executive Function Scale (AEFS; Huang et al., 2014), a 21-item self-report instrument that taps three dimensions: inhibitory control (6 items; e.g., “I am impulsive”), cognitive flexibility (8 items; e.g., “I am bothered by changes of plans”), and working memory (7 items; e.g., “I forget ongoing tasks”). Each item is scored from 1 (never) to 3 (often), with higher scores reflecting poorer executive function. Cronbach’s α = 0.95 in the current sample. The complete item-to-subscale mapping with descriptive statistics is provided in Table S1.

### 2.3. Statistical Analysis

Descriptive statistics (means and standard deviations) for the three EF dimensions and the 19 NSSI function items were computed using SPSS 25.0. Subsequent network analyses were performed in R (version 4.11).

Prior to network estimation, potential node redundancy was assessed using the goldbricker function in the R package networktools (version 1.5.1; P. Jones, 2022). Although several pairs of nodes showed moderate to high bivariate correlations, no pair exceeded 0.90, suggesting that node redundancy was not a critical concern. A Gaussian graphical model (GGM) was used to estimate a regularized partial correlation network (Epskamp & Fried, 2018). The model was fitted via the graphical LASSO algorithm (Friedman et al., 2008) with the Extended Bayesian Information Criterion (EBIC; Foygel & Drton, 2010) for tuning parameter selection, yielding a sparse and readily interpretable network. In the resulting network, each variable is represented as a node, and associations between nodes are represented as edges. The layout was determined by the Fruchterman–Reingold algorithm (Fruchterman & Reingold, 1991), which places strongly connected nodes near the center and weakly connected nodes at the periphery. Edges were colored blue for positive partial correlations and red for negative ones, with edge thickness proportional to the magnitude of the correlation. Network estimation and visualization relied on the R package qgraph (version 1.9.8; Epskamp et al., 2012).

To identify highly influential nodes, expected influence (EI) was calculated for each node. EI is defined as the sum of the edge weights linking that node to all other nodes in the network (Robinaugh et al., 2016). Bridge expected influence (BEI) was computed using the networktools package (P. J. Jones et al., 2021) to quantify the role of nodes in connecting the executive function and NSSI function communities. BEI equals the sum of edge weights between a given node and all nodes belonging to a different community (P. J. Jones et al., 2021).

The accuracy and stability of the network were evaluated with the bootnet package (Epskamp et al., 2018). First, non-parametric bootstrapping (1000 bootstrap samples) was performed to construct 95% confidence intervals for the edge weights, providing an estimate of edge weight precision. Second, case dropping bootstrap procedures (1000 bootstrap samples) were used to assess the stability of EI and BEI, yielding the correlation stability (CS) coefficient. CS values above 0.50 indicate excellent stability and should not fall below 0.25. Finally, bootstrapped difference tests (1000 bootstrap samples, α = 0.05) were conducted to determine whether edge weights and centrality indices (EI and BEI) differed significantly across nodes.

## 3. Results

### 3.1. Demographic Characteristics and Descriptive Statistics

A total of 1078 participants (82.6% female, Mage = 19.07, SD = 1.03) who had engaged in at least one NSSI behavior in the past year were included. Means, standard deviations, EI, and BEI for the three executive function dimensions and the 19 NSSI function items are presented in Table 1.

### 3.2. Network Structure

**Figure 1:**
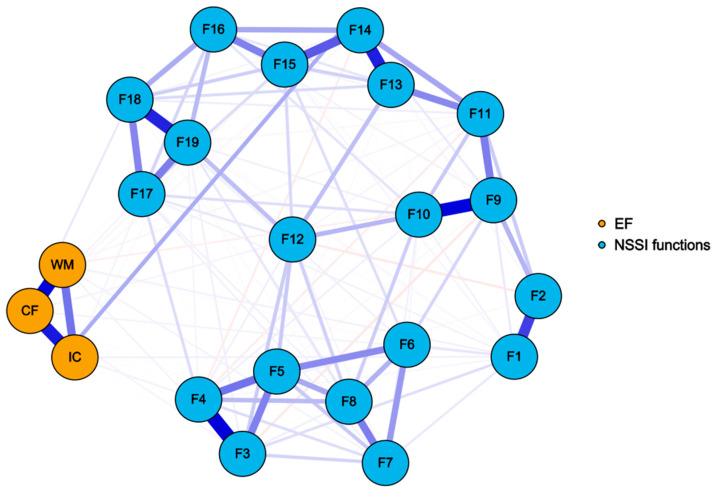
Network structure of EF dimensions and NSSI functions in university students with NSSI. Nodes represent EF dimensions and NSSI functions. Blue edges indicate positive associations and orange edges indicate negative associations between nodes. Because all negative edges in the present network are very weak in magnitude, ranging from −0.01 to −0.04, their orange coloration is extremely faint and may appear light orange or nearly white. Edge thickness reflects the strength of the association, with thicker edges representing stronger partial correlations. EF: executive function; NSSI: non-suicidal self-injury.

The regularized partial correlation network comprised 22 nodes (three executive function dimensions and 19 NSSI function items). Of the 231 possible edges, 117 (50.65%) were non-zero, indicating substantial interconnectivity among the variables (Figure 1). Within the executive function community, the strongest connection occurred between cognitive flexibility (CF) and working memory (WM) (edge weight = 0.46), reflecting their close coordination in supporting adaptive cognitive processes. Within the NSSI function community, the strongest edge linked “coping with sadness and disappointment” (F3) and “expressing despair and hopelessness” (F4) (weight = 0.48), suggesting that motivations to manage sadness and to communicate hopelessness frequently co-occur as a cluster of emotional distress functions. Across communities, the most prominent cross-community edge was between inhibitory control and “having a desire to harm myself and cannot stop” (F14) (weight = 0.16), indicating that poorer inhibitory control was directly associated with a more intense, uncontrollable urge to self-injure. All edge weights are provided in Table S2 in the Supplementary Materials. The raw zero-order Pearson correlation matrix among all 22 nodes is provided in Table S3 in the Supplementary Materials.

### 3.3. Centrality Analysis

**Figure 2:**
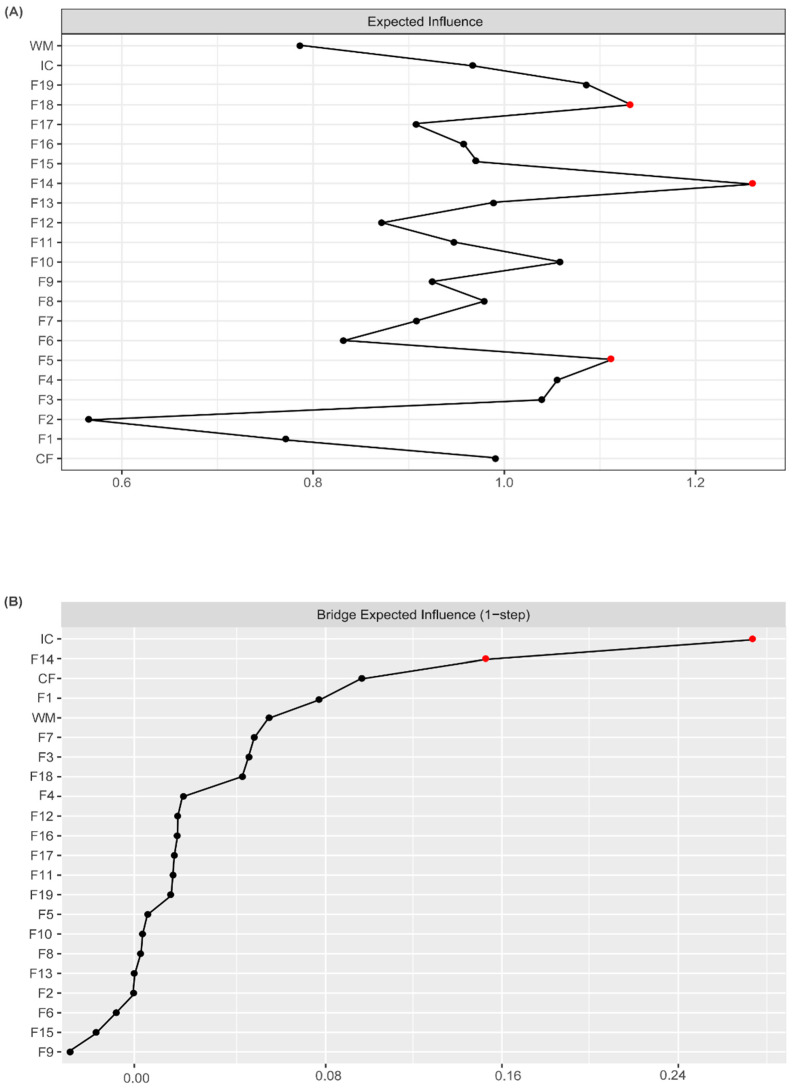
Centrality indices and bridge centrality indices of each node in the present network (raw values). (**A**) Expected influence. Red nodes indicate the three nodes with the highest EI values, representing the most central nodes in the network. (**B**) Bridge expected influence. Red nodes indicate the two bridge nodes with the highest BEI values, con-necting the EF and NSSI function communities. See Table 1 for the item descriptions corresponding to each node. Ab-breviations: IC, Inhibitory control; CF, Cognitive flexibility; WM, Working memory. F1–F19 correspond to the NSSI function items listed in Table 1.

EI was computed to identify the most influential nodes in the overall network (Figure 2A). The three highest EI values were observed for “having a desire to harm myself and cannot stop” (F14), “letting others make changes” (F18), and “self-punishment” (F5) (EI = 1.26, 1.13, 1.11, respectively). These findings suggest that an uncontrollable urge to self-injure, interpersonal influence, and self-punishment motives may function as central mechanisms that sustain or propagate NSSI in young people, and could therefore serve as potential clinical markers for early detection and intervention.

### 3.4. Bridge Centrality Analysis

As shown in Figure 2B, the BEI of each node in the executive function–NSSI function network is displayed. Inhibitory control showed the highest BEI value in the executive function community (BEI = 0.28), and “having a desire to harm myself and cannot stop” (F14) showed the highest BEI value in the NSSI function community (BEI = 0.14). Therefore, inhibitory control and “having a desire to harm myself and cannot stop” (F14) were regarded as the bridge nodes connecting the two communities.

### 3.5. Network Accuracy and Stability

The bootstrapped 95% CIs were relatively narrow (see Supplementary Figure S1), indicating that the edge weights were accurate and stable. The bootstrapped difference tests for edge weights are shown in the Supplementary Materials (Supplementary Figure S2). The CS coefficients of EI and BEI were both 0.75, and thus above 0.50, indicating that both EI and BEI had excellent stability (see Figures S3 and S4). The bootstrapped difference tests showed that the EI values of the identified central nodes were significantly different from those of most other nodes (see Supplementary Figure S5). For BEI, most pairwise comparisons were not statistically significant (see Supplementary Figure S6).

## 4. Discussion

This study is the first to apply network analysis to examine associations between specific EF dimensions and individual NSSI functions among Chinese university students with past-year NSSI. The findings reveal underlying connectivity patterns, identify central and bridge nodes, and highlight potential clinical implications. Importantly, although “inhibitory control” (IC) and “having a desire to harm myself and cannot stop” (F14) emerged as key bridge nodes, the cross-sectional design precludes causal inference; all results should be interpreted as observed associations rather than direct causal effects.

The network comprised 231 possible edges, with 117 (50.65%) exhibiting non-zero weights, indicating a densely interconnected system of executive function dimensions and NSSI functions. This substantial interconnectivity suggests that cognitive deficits and self-injurious motivations are not isolated phenomena but components of a mutually reinforcing system, consistent with the cognitive–emotional model of NSSI (Hasking et al., 2017). Although these findings support the potential value of integrated interventions targeting both cognitive and motivational processes, longitudinal studies are required to clarify the temporal directionality of these associations.

Within the EF community, the strongest connection occurred between “cognitive flexibility” (CF) and “working memory” (WM) (edge weight = 0.46), reflecting the coordinated interplay between the ability to shift mental sets and the capacity to actively maintain and update relevant information (Diamond, 2013; Miyake et al., 2000). This strong coupling suggests that these two cognitive processes are tightly interdependent: cognitive flexibility may rely on working memory to temporarily hold alternative strategies in mind while disengaging from a dominant but maladaptive response. When distressed, individuals with weakened CF–WM coordination may struggle not only to generate adaptive alternatives but also to keep those alternatives active long enough to guide behavior away from self-injury. This interpretation is supported by converging evidence from neuropsychological studies. On the one hand, adolescents engaging in NSSI have been found to exhibit working memory deficits on spatial working memory tasks (Fikke et al., 2011). On the other hand, among depressed adolescents, those with NSSI demonstrate significantly poorer cognitive flexibility compared to those without NSSI, and cognitive flexibility further declines as NSSI frequency increases (Y. Wang et al., 2023). More broadly, prior work has indicated that executive function deficits may be associated with emotion regulation difficulties that contribute to the maintenance of self-injurious behavior (Bredemeier & Miller, 2015). Nevertheless, given the reliance on self-reported measures, potential measurement bias should be acknowledged, and future studies incorporating performance-based neurocognitive tasks are warranted to verify whether this CF–WM coupling reflects actual cognitive performance.

Similarly, within the NSSI function community, the strongest edge linked “coping with sadness and disappointment” (F3) and “expressing despair and hopelessness” (F4) (edge weight = 0.48). This indicates a functional clustering of emotional distress functions, suggesting that motivations to manage sadness and to communicate hopelessness frequently co-occur. These processes may reflect a broader pattern in which NSSI serves both intrapersonal emotion regulation and interpersonal communication functions simultaneously, consistent with prior research showing that individuals who engage in NSSI rarely endorse a single function (Coppersmith et al., 2021; Klonsky, 2011). A meta-analysis confirmed that intrapersonal functions are the most commonly endorsed (Taylor et al., 2018), and a recent network study further identified affect regulation and self-punishment as core intrapersonal functions directly linked to NSSI persistence (Szewczuk-Bogusławska et al., 2024). The co-occurrence of these emotional distress functions may create a particularly reinforcing cycle, wherein NSSI is perceived as effective for both relieving internal distress and expressing emotional pain to others, thereby increasing the likelihood of repeated engagement. This pattern aligns directly with the four-function model, which posits that automatic and social reinforcement processes can operate simultaneously to maintain the behavior (Nock & Prinstein, 2004).

Beyond the within-community connections, cross-community analysis revealed that inhibitory control (IC) was directly associated with “having a desire to harm myself and cannot stop” (F14), representing the strongest bridge between the two communities. This suggests that poorer inhibitory control is specifically linked to a more intense, uncontrollable urge to self-injure, aligning with the cognitive–emotional model of NSSI (Hasking et al., 2017). Empirical research has consistently linked inhibitory control deficits to self-injurious behavior (McHugh et al., 2019), with greater response inhibition deficits found in more severe NSSI (Fikke et al., 2011), event-related potential evidence demonstrating impaired behavioral inhibitory control in adolescents with depression and NSSI (Ma et al., 2023), and weak inhibitory control independently predicting suicide risk (Venables et al., 2015). The specific association between inhibitory control and the uncontrollable urge to self-injure, rather than other NSSI functions, suggests a targeted pathway: inhibitory deficits may not broadly increase all NSSI motivations, but rather specifically amplify the experience of losing control over self-injurious impulses. Clinically, this indicates that interventions enhancing inhibitory control may be particularly beneficial for young adults who experience their NSSI as uncontrollable. However, these interpretations should be considered with caution; the cross-sectional design cannot determine whether inhibitory deficits precede or follow the development of uncontrollable urges.

Bridge centrality analysis identified “inhibitory control” (IC) from the EF community and “having a desire to harm myself and cannot stop” (F14) from the NSSI function community as the bridge nodes connecting the two communities. Inhibitory control showed the highest bridge centrality, suggesting that this dimension plays a particularly important role in linking cognitive deficits to NSSI functions. This aligns with neuropsychological research showing that deficits in inhibition-related executive functions are among the most robust cognitive correlates of NSSI (Fikke et al., 2011). This finding does not imply direct causality; instead, it highlights inhibitory control as the cognitive mechanism most strongly associated with NSSI motivations and underscores its potential role in connecting cognitive and behavioral processes. Previous research has suggested that central and bridge nodes represent promising targets for intervention because modifying them may disrupt the spread of activation across the network (Robinaugh et al., 2016; P. J. Jones et al., 2021; Jiang et al., 2026; S. Wang et al., 2026). Consequently, interventions that enhance inhibitory control may be especially crucial for individuals experiencing their NSSI as uncontrollable. Future research could explore whether specifically targeting the pathway linking inhibitory control with the uncontrollable urge yields clinical benefits. However, these exploratory intervention strategies require further empirical validation to establish their clinical efficacy.

Beyond bridge centrality, we also examined overall node centrality using EI. This analysis identified “having a desire to harm myself and cannot stop” (F14) as the node with the highest EI, indicating that the uncontrollable urge to self-injure occupies a central position within the network and is broadly connected to both executive function dimensions and other NSSI functions, consistent with prior network findings (Guan et al., 2024). Notably, as noted earlier, F14 also emerged as a key bridge node. This dual role—serving as both the most central node and a bridge between communities—suggests that this experience may function as a critical hub through which cognitive deficits and self-injurious motivations mutually reinforce one another. Although this connectivity pattern is suggestive, it remains tentative; further longitudinal research is needed to clarify whether changes in this node affect the broader network. “Letting others make changes” (F18) and “self-punishment” (F5) also showed high EI, suggesting that interpersonal influence and self-punishment motives may function as additional central mechanisms sustaining NSSI. The prominence of self-punishment aligns with research showing that it is a common function of self-injury independently associated with elevated suicide attempt risk (Klonsky, 2007; Y. Shen et al., 2023). The high centrality of interpersonal influence underscores that NSSI in young adults is not solely an intrapersonal phenomenon but also serves communicative functions within social contexts, consistent with the social reinforcement dimension of the four-function model (Nock, 2009). Taken together, these centrality patterns support a personalized approach to intervention. For individuals who use NSSI as self-punishment, compassion-focused therapy (targeting self-criticism and shame) may be particularly beneficial (van Vliet & Kalnins, 2011). For those endorsing affect regulation functions (e.g., F3, F4), dialectical behavior therapy or emotion regulation group therapy may be optimal (Linehan & Wilks, 2015; Andover & Morris, 2014). More broadly, given that inhibitory control bridges cognitive deficits with the uncontrollable urge to self-injure, interventions that directly enhance inhibitory control (e.g., cognitive training or mindfulness-based approaches) could be integrated for individuals reporting high levels of F14. These observations support the rationale to personalize therapeutic plans according to the specific functions endorsed by individuals with NSSI.

Despite providing novel insights, this study has several limitations. First, the cross-sectional design precludes causal inference. Future studies should adopt longitudinal designs with multiple time points to clarify the dynamic trajectories and potential causal mechanisms linking executive function to NSSI. Second, the participants were recruited exclusively from several universities across four provincial-level administrative divisions in China (Zhejiang, Shaanxi, and Hunan Provinces, and Shanghai Municipality)**.** Although the sample included young adults from different regions, all were university students, and the majority were female (82.6%), which may limit generalizability. This gender imbalance likely reflects the underlying epidemiology of NSSI. Epidemiological evidence and meta-analytic findings consistently indicate that NSSI is more prevalent among female adolescents and young adults, a pattern documented across many cultural settings, particularly in North America and Europe (Bresin & Schoenleber, 2015; Fox et al., 2018; Moloney et al., 2024). In a prior study of Chinese adolescents with depression conducted by our group, females showed a significantly higher prevalence of NSSI than males (77.82% vs. 57.45%; G. Shen et al., 2024), further corroborating this gender difference in the Chinese clinical context. Additionally, it is possible that men may be less willing to disclose emotional distress and participate in mental health surveys due to traditional masculine norms (Qiu et al., 2024), and the gender composition of the universities from which we recruited, especially in certain humanities and social science programs, may also have contributed to the skewed distribution. It should be noted that the gender pattern observed in NSSI behavior (higher prevalence among females) is distinct from the gender paradox in suicide mortality (higher death rates among males), and our findings pertain only to NSSI functions among living individuals. These explanations remain speculative; therefore, readers should exercise caution when generalizing these findings to male populations. Future studies should replicate these analyses in community-based, clinical, and cross-cultural samples and purposefully oversample male participants to examine potential gender differences in the EF–NSSI function network. Third, all variables were assessed via self-reported questionnaires, which may be subject to recall bias, underreporting, and social desirability effects. Furthermore, self-report measures of executive function evaluate the subjective perception of executive difficulties, which can be heavily influenced by current depressive states, and may not fully capture actual cognitive performance (Diamond, 2013). The reliance on self-report rather than performance-based neurocognitive tasks represents an important limitation, as perceived executive dysfunction and objectively measured executive function may reflect distinct constructs. Future studies should integrate multi-method assessments such as performance-based neurocognitive tasks and ecological momentary assessment to more accurately capture both subjective and objective aspects of executive functioning. Fourth, the network model included only executive function dimensions and NSSI functions, omitting other important correlates. Among these, depressive symptoms represent a particularly important missing variable, given their well-established associations with both executive dysfunction and NSSI (Y. Shen et al., 2023). Other unmeasured variables, such as emotion regulation, trauma exposure, and severity or intensity of NSSI, may also affect executive functions and confound the observed network edges. Future studies should consider building more complex, multidimensional network models that integrate these psychosocial and clinical factors. Finally, the study was conducted in a single cultural context. Cultural norms and stigma associated with mental health in Chinese populations may differ substantially from those in Western populations (Qu et al., 2023); therefore, caution is warranted when generalizing these findings to young adults from other cultural contexts.

## 5. Conclusions

This is the first study to investigate the network structure of executive function dimensions and non-suicidal self-injury (NSSI) functions in Chinese university students with past-year NSSI using network analysis. This network analysis revealed that inhibitory control (IC) served as a core bridge node linking executive dysfunction with NSSI functions, while “having a desire to harm myself and cannot stop” (F14) emerged as a dual-role node, being both the most influential NSSI function and a key bridge node. “Letting others make changes” (F18) and “self-punishment” (F5) also showed high centrality. These findings highlight inhibitory control (IC), the uncontrollable urge to self-injure (F14), letting others make changes (F18), and self-punishment (F5) as key connectors and central drivers between cognitive deficits and self-injurious motivations, providing potential targets for future intervention. Although the cross-sectional design and reliance on self-reports warrant cautious interpretation, these results offer a basis for developing targeted cognitive–behavioral strategies to disrupt the maladaptive cycle maintaining NSSI in this population.

## Figures and Tables

**Table 1:** The means, standard deviations, EI, and BEI for NSSI function items and executive function dimensions.

Variables	M ± SD	EI	BEI
NSSI function items			
F1: Express anger	2.16 ± 1.07	0.77	0.08
F2: Avoiding things I dislike or that make me unhappy	2.33 ± 1.22	0.56	0.00
F3: Coping with sadness and disappointment	1.76 ± 0.91	1.03	0.05
F4: Expressing despair and hopelessness	1.68 ± 0.87	1.06	0.02
F5: Self-punishment	1.84 ± 0.99	1.11	0.01
F6: Relieve stress or anxiety	2.00 ± 1.09	0.83	−0.01
F7: Control myself to calm down	1.95 ± 1.03	0.90	0.05
F8: Helping myself stop negative thoughts	1.82 ± 0.97	0.98	0.00
F9: Escaping from numbness and feeling unreal	2.19 ± 1.13	0.92	−0.03
F10: Making myself feel less lonely	2.07 ± 1.01	1.06	0.00
F11: Bringing happiness, enjoyment, and making myself feel good	2.57 ± 1.23	0.95	0.02
F12: Attracting others’ attention	1.99 ± 1.05	0.87	0.02
F13: Because my friends have done this before	2.46 ± 1.21	0.99	0.00
F14: Having a desire to harm myself and cannot stop	2.39 ± 1.18	1.26	0.14
F15: Avoiding harming others	2.21 ± 1.12	0.97	−0.02
F16: Protecting myself from others’ attacks	2.35 ± 1.15	0.96	0.02
F17: Taking revenge on others	2.05 ± 0.99	0.90	0.02
F18: Letting others make changes	2.30 ± 1.15	1.13	0.04
F19: Gaining understanding from others	2.09 ± 1.05	1.09	0.02
Executive function dimensions			
IC: Inhibitory control	8.69 ± 2.43	0.97	0.28
CF: Cognitive flexibility	12.09 ± 3.56	0.99	0.10
WM: Working memory	10.82 ± 3.29	0.79	0.06

Note: M, mean; SD, standard deviation; EI, expected influence. BEI, bridge expected influence.

## Data Availability

The datasets presented in this article are not readily available because the datasets involve unfinished research projects. If necessary, requests to access the datasets should be directed to the corresponding authors.

## Supplementary Materials

The following supporting information can be downloaded at: https://www.mdpi.com/article/10.3390/bs16071156/s1, Figure S1: Accuracy of edge weights in the network; Figure S2: Bootstrapped difference test for edge weights in the network; Figure S3: Stability of node expected influences in the network; Figure S4: Stability of node bridge expected influences in the network; Figure S5: Bootstrapped difference test for node expected influences in the network; Figure S6: Bootstrapped difference test for node bridge expected influences in the network; Table S1: Item-Level Mapping and Descriptive Statistics for the Adolescent Executive Function Scale (AEFS); Table S2: The edge weights within the EF-NSSI function network; Table S3: Raw Zero-Order Bivariate Correlation Matrix Among All Network Nodes in the Analytic Sample (N = 1078).
