# Supplementary material for: Are Specific Components of Executive Function Associated with the Functions of Non-Suicidal Self-Injury? A Network Analysis of Chinese University Students with Past-Year NSSI
Source: Behav Sci (Basel). 2026 Jul 9;16(7):1156. doi: 10.3390/bs16071156 (PMC13405853; doi:10.3390/bs16071156)
Supplement: Supplementary file 1 [file behavsci-16-01156-s001.zip › behavsci-4359365-supplementary.pdf]

# Supplementary materials

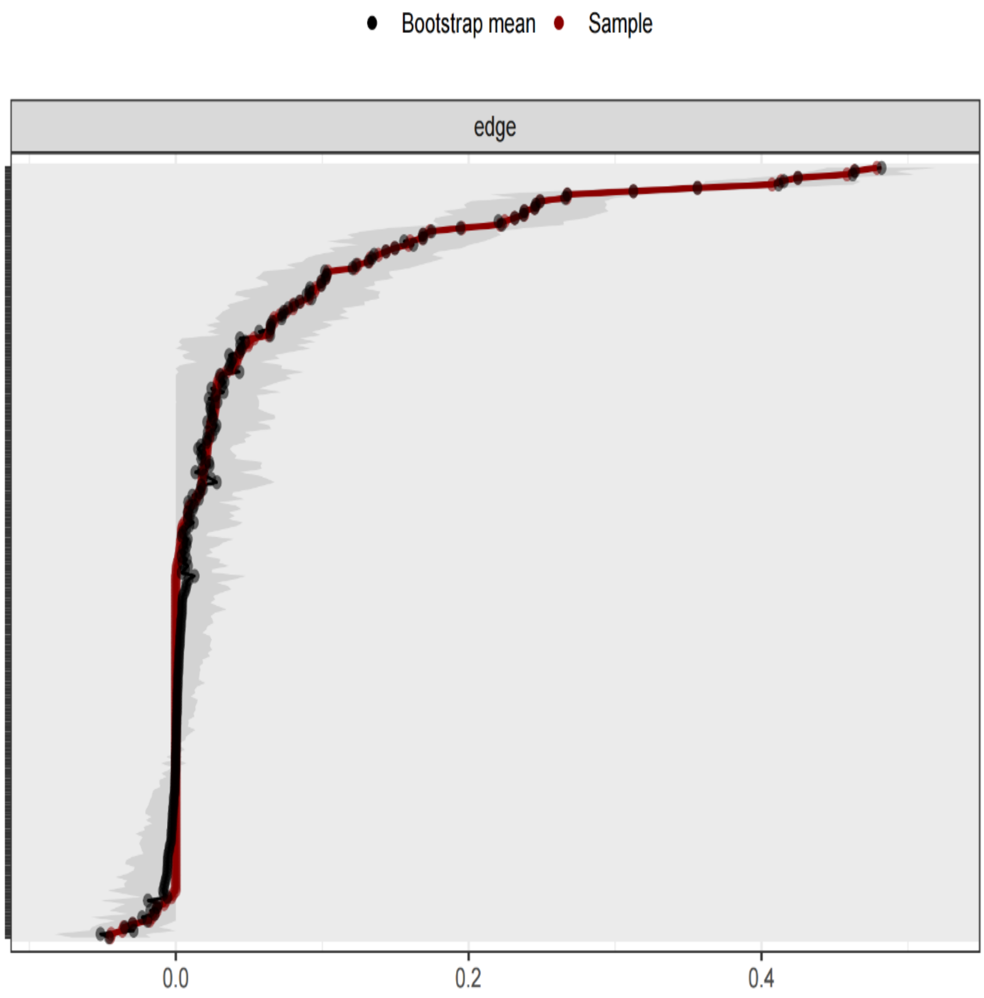


Supplementary Figure S1 Accuracy of edge weights in the network.

*Note*: The red line depicts the sample edge weights and the gray bar depicts the bootstrapped confidence interval.

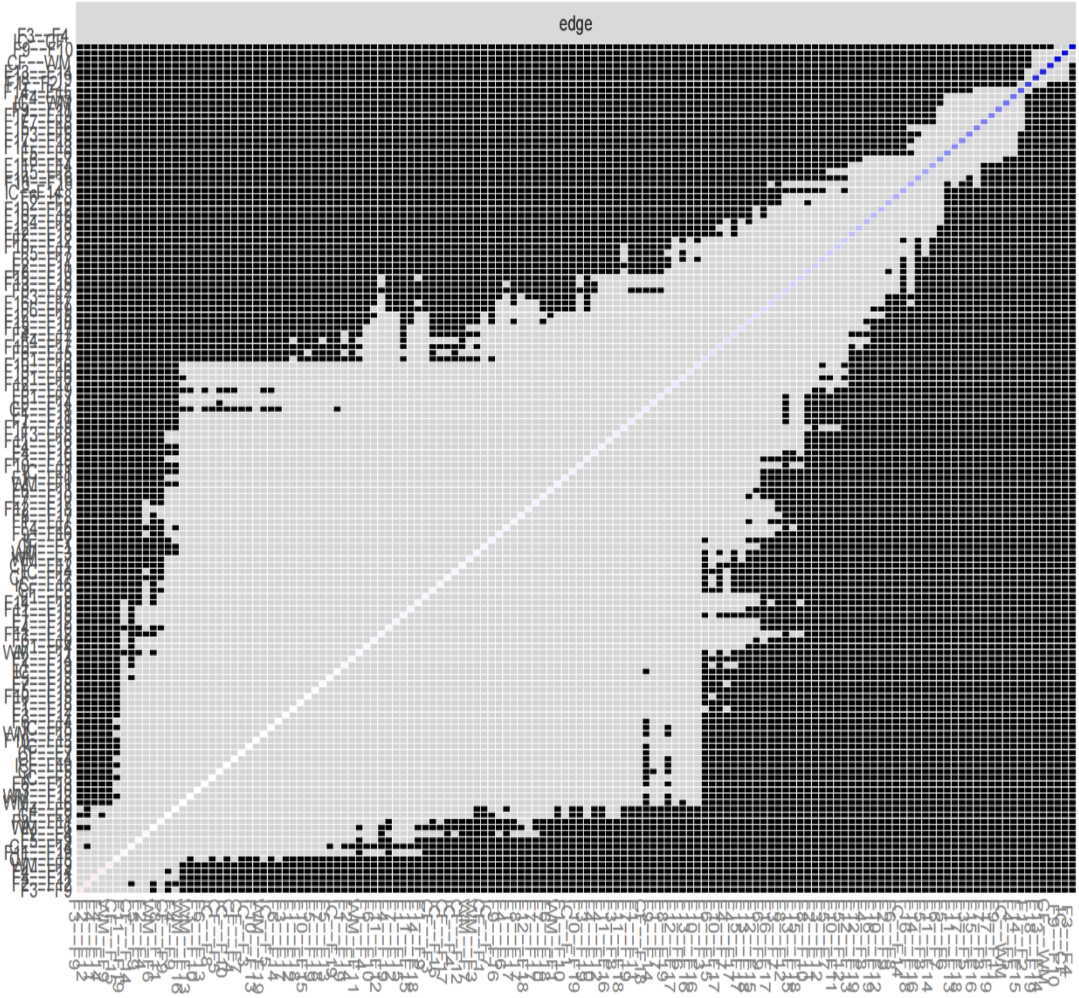


Supplementary Figure S2 Bootstrapped difference test for edge weights in the network. *Note*: Gray boxes indicate edge weights that do not differ significantly from one another, while black boxes indicate edge weights that do differ significantly. Blue and red boxes on the diagonal correspond to edge weights with positive and negative correlations, respectively.

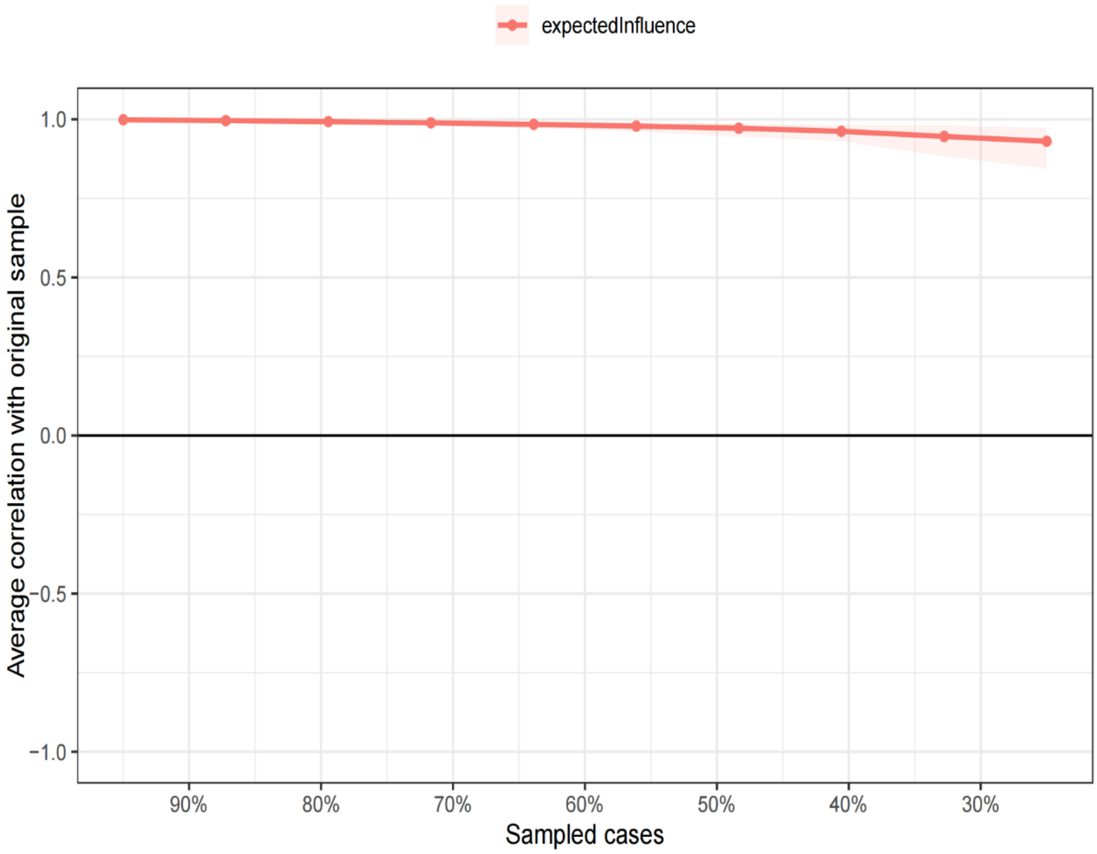


Supplementary Figure S3 Stability of node expected influences in the network.

*Note:* The red bar represents the average correlation between node expected influences in the full sample and subsample with the red area depicting the 2.5th quantile to the 97.5th quantile.

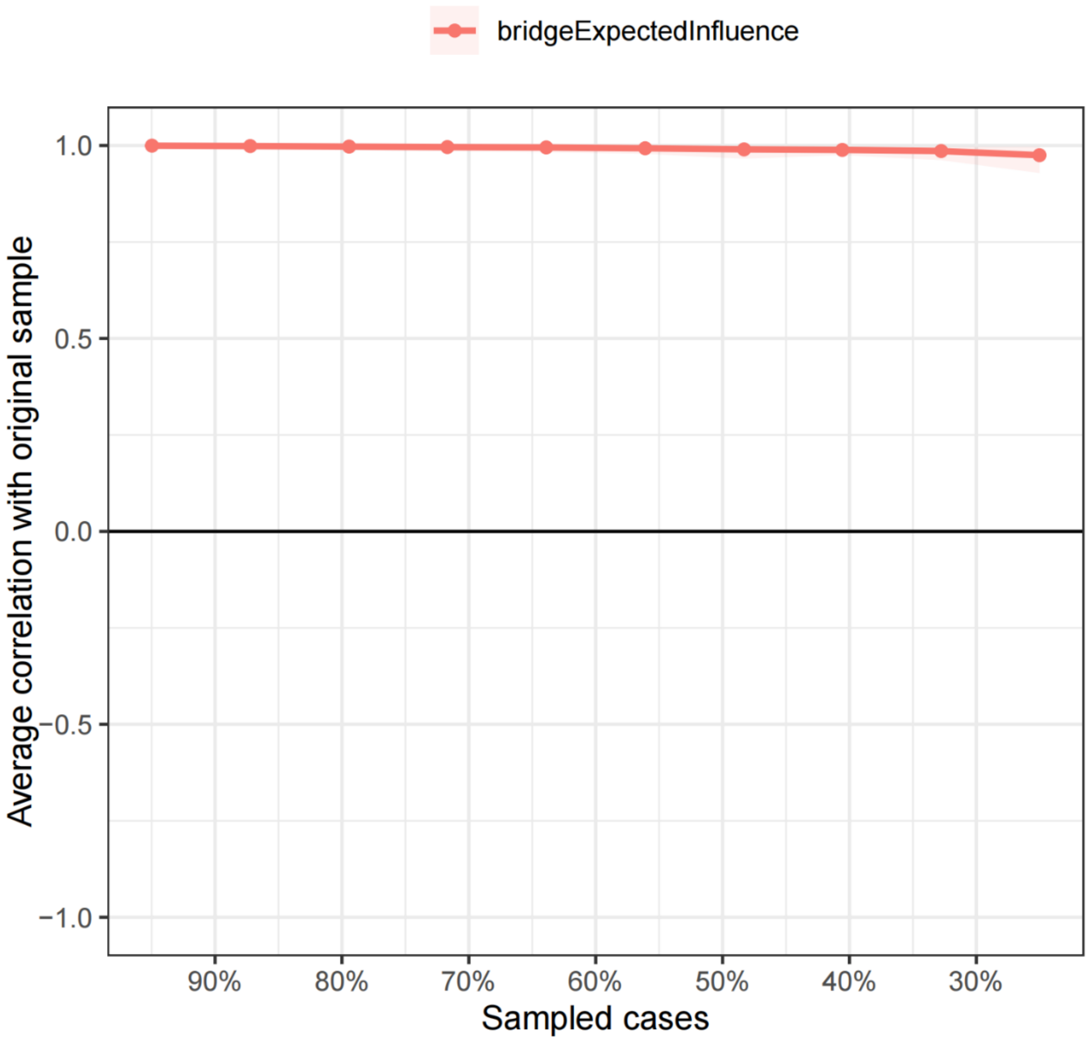


Supplementary Figure S4 Stability of node bridge expected influences in the network.

*Note:* The red bar represents the average correlation between node bridge expected influences in the full sample and subsample with the red area depicting the 2.5th quantile to the 97.5th quantile.

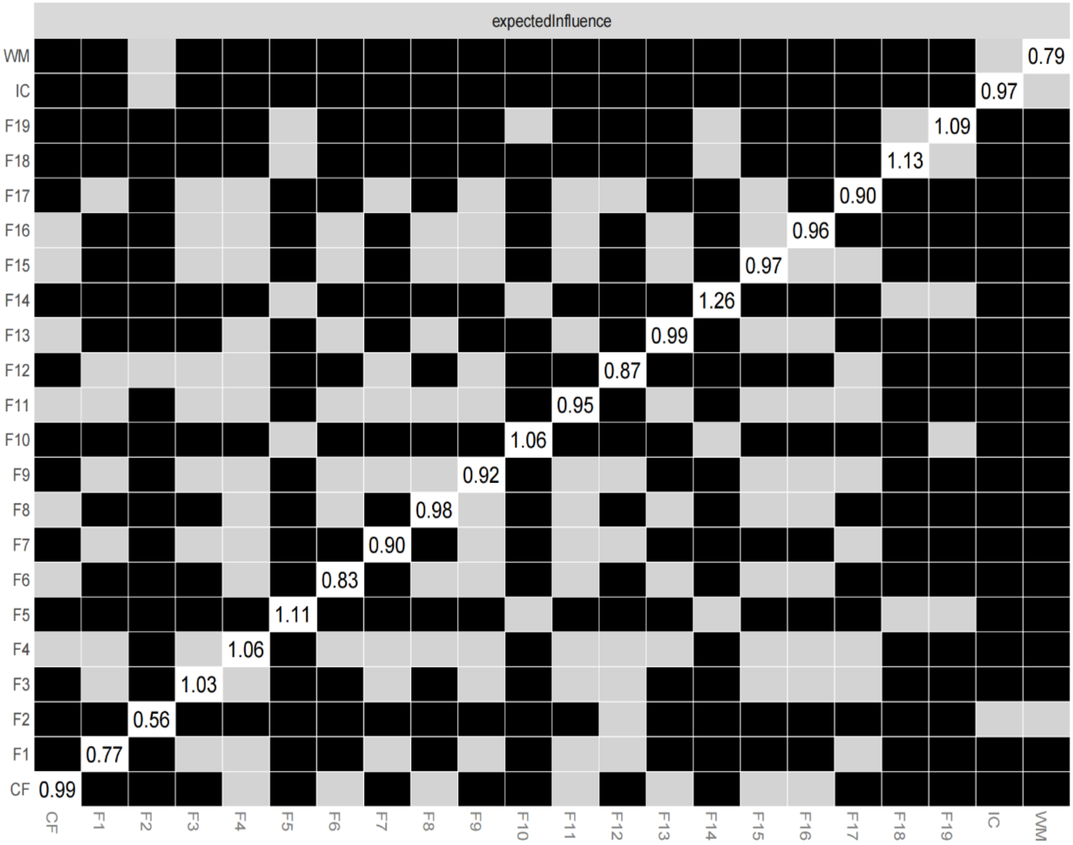


Supplementary Figure S5 Bootstrapped difference test for node expected influences in the network.

*Note:* Gray boxes indicate node expected influences that do not differ significantly from one another, while black boxes indicate node expected influences that do differ significantly. The numbers in the white boxes (i.e., diagonal line) represent the values of node expected influences.

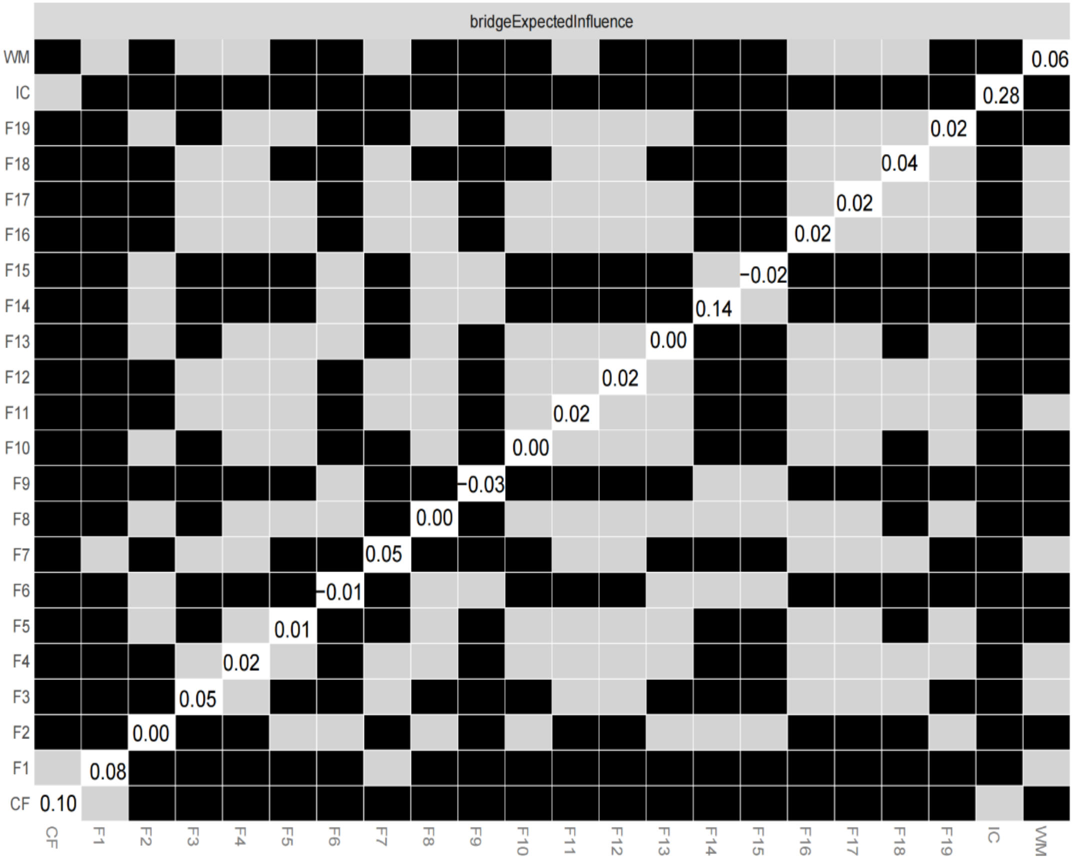


Supplementary Figure S6 Bootstrapped difference test for node bridge expected influences in the network.

*Note:* Gray boxes indicate node bridge expected influences that do not differ significantly from one another, while black boxes indicate node bridge expected influences that do differ significantly. The numbers in the white boxes (i.e., diagonal line) represent the values of node bridge expected influences.

**Table S1.** Item-Level Mapping and Descriptive Statistics for the Adolescent Executive Function Scale (AEFS)

| Item | Subscale | M | SD |
|---|---|---|---|
| IC1: Impulsive | IC | 1.56 | 0.55 |
| IC2: Overreactive | IC | 1.47 | 0.54 |
| IC3: Inappropriate talking | IC | 1.45 | 0.53 |
| IC4: Acting without thinking | IC | 1.44 | 0.55 |
| IC5: Speaking without thinking | IC | 1.50 | 0.55 |
| IC6: Wild/uncontrolled behavior | IC | 1.27 | 0.47 |
| CF1: Bothered by changes of plans | CF | 1.70 | 0.63 |
| CF2: Bothered by unexpected events | CF | 1.51 | 0.58 |
| CF3: Bothered by dealing with changes | CF | 1.39 | 0.56 |
| CF4: Difficulty solving problems creatively | CF | 1.45 | 0.57 |
| CF5: Trouble adapting | CF | 1.48 | 0.58 |
| CF6: Difficulty switching tasks | CF | 1.45 | 0.56 |
| CF7: Difficulty generating alternative solutions | CF | 1.66 | 0.62 |
| CF8: Difficulty considering alternatives | CF | 1.48 | 0.57 |
| WM1: Forgetting ongoing tasks | WM | 1.66 | 0.62 |
| WM2: Forgetting assigned items | WM | 1.48 | 0.57 |
| WM3: Losing track of things | WM | 1.62 | 0.62 |
| WM4: Forgetting instructions | WM | 1.54 | 0.58 |
| WM5: Forgetting to submit work | WM | 1.51 | 0.57 |
| WM6: Forgetting to bring items | WM | 1.47 | 0.56 |
| WM7: Always losing things | WM | 1.54 | 0.60 |

*Note.* IC = Inhibitory Control; CF = Cognitive Flexibility; WM = Working Memory. Items are rated on a 3-point scale: 1 = never, 2 = sometimes, 3 = often. Higher scores indicate poorer executive function.

**Table S2.** The edge weights within the EF- NSSI function network

| | IC | CF | WM | F1 | F2 | F3 | F4 | F5 | F6 | F7 | F8 | F9 | F10 | F11 | F12 | F13 | F14 | F15 | F16 | F17 | F18 | F19 |
|---|---|---|---|---|---|---|---|---|---|---|---|---|---|---|---|---|---|---|---|---|---|---|
| IC | | 0.42 | 0.27 | 0.03 | 0.00 | 0.00 | 0.02 | 0.00 | 0.00 | 0.02 | 0.00 | 0.00 | 0.00 | 0.00 | 0.00 | 0.00 | 0.16 | 0.00 | 0.02 | 0.00 | 0.00 | 0.01 |
| CF | 0.42 | | 0.46 | 0.02 | 0.00 | 0.02 | 0.00 | 0.00 | 0.00 | 0.00 | 0.00 | 0.00 | 0.00 | 0.00 | 0.02 | 0.00 | -0.01 | -0.02 | 0.00 | 0.02 | 0.04 | 0.00 |
| WM | 0.27 | 0.46 | | 0.03 | 0.00 | 0.02 | 0.00 | 0.00 | -0.01 | 0.02 | 0.00 | -0.03 | 0.00 | 0.02 | 0.00 | 0.00 | 0.00 | 0.00 | 0.00 | 0.00 | 0.00 | 0.00 |
| F1 | 0.03 | 0.02 | 0.03 | | 0.36 | 0.06 | 0.02 | 0.00 | 0.05 | 0.04 | 0.00 | 0.02 | 0.03 | 0.04 | 0.01 | 0.00 | 0.00 | 0.02 | 0.00 | 0.02 | 0.01 | 0.01 |
| F2 | 0.00 | 0.00 | 0.00 | 0.36 | | 0.00 | -0.01 | 0.00 | 0.00 | 0.00 | 0.00 | 0.14 | 0.00 | 0.10 | -0.04 | 0.00 | 0.01 | 0.00 | 0.00 | 0.00 | 0.00 | 0.00 |
| F3 | 0.00 | 0.02 | 0.02 | 0.06 | 0.00 | | 0.48 | 0.24 | 0.00 | 0.09 | 0.03 | -0.04 | 0.00 | 0.00 | 0.10 | 0.00 | 0.00 | 0.00 | 0.00 | 0.00 | 0.00 | 0.03 |
| F4 | 0.02 | 0.00 | 0.00 | 0.02 | -0.01 | 0.48 | | 0.27 | 0.02 | 0.07 | 0.13 | 0.00 | 0.03 | -0.04 | 0.03 | 0.00 | -0.04 | 0.00 | 0.00 | 0.07 | 0.00 | 0.02 |
| F5 | 0.00 | 0.00 | 0.00 | 0.00 | 0.00 | 0.24 | 0.27 | | 0.22 | 0.10 | 0.17 | -0.01 | 0.00 | 0.00 | 0.10 | 0.01 | 0.00 | 0.00 | 0.00 | 0.00 | 0.00 | 0.01 |
| F6 | 0.00 | 0.00 | -0.01 | 0.05 | 0.00 | 0.00 | 0.02 | 0.22 | | 0.20 | 0.15 | 0.08 | 0.02 | 0.00 | 0.00 | 0.00 | 0.00 | 0.06 | 0.00 | 0.00 | 0.03 | 0.00 |
| F7 | 0.02 | 0.00 | 0.02 | 0.04 | 0.00 | 0.09 | 0.07 | 0.10 | 0.20 | | 0.24 | 0.00 | 0.03 | 0.00 | 0.02 | 0.00 | 0.00 | 0.00 | 0.00 | 0.03 | 0.01 | 0.04 |
| F8 | 0.00 | 0.00 | 0.00 | 0.00 | 0.00 | 0.03 | 0.13 | 0.17 | 0.15 | 0.24 | | 0.00 | 0.10 | -0.01 | 0.09 | 0.00 | 0.00 | 0.00 | 0.00 | 0.02 | 0.00 | 0.04 |
| F9 | 0.00 | 0.00 | -0.03 | 0.02 | 0.14 | -0.04 | 0.00 | -0.01 | 0.08 | 0.00 | 0.00 | | 0.46 | 0.25 | 0.00 | 0.00 | 0.04 | 0.00 | 0.02 | 0.00 | 0.00 | 0.00 |
| F10 | 0.00 | 0.00 | 0.00 | 0.03 | 0.00 | 0.00 | 0.03 | 0.00 | 0.02 | 0.03 | 0.10 | 0.46 | | 0.10 | 0.14 | 0.00 | 0.00 | 0.01 | 0.05 | 0.06 | 0.00 | 0.03 |
| F11 | 0.00 | 0.00 | 0.02 | 0.04 | 0.10 | 0.00 | -0.04 | 0.00 | 0.00 | 0.00 | -0.01 | 0.25 | 0.10 | | 0.02 | 0.22 | 0.17 | 0.02 | 0.03 | 0.00 | 0.03 | -0.02 |
| F12 | 0.00 | 0.02 | 0.00 | 0.01 | -0.04 | 0.10 | 0.03 | 0.10 | 0.00 | 0.02 | 0.09 | 0.00 | 0.14 | 0.02 | | 0.12 | 0.00 | 0.08 | 0.00 | 0.05 | 0.03 | 0.12 |
| F13 | 0.00 | 0.00 | 0.00 | 0.00 | 0.00 | 0.00 | 0.00 | 0.01 | 0.00 | 0.00 | 0.00 | 0.00 | 0.00 | 0.22 | 0.12 | | 0.41 | 0.09 | 0.05 | 0.00 | 0.07 | 0.00 |
| F14 | 0.16 | -0.01 | 0.00 | 0.00 | 0.01 | 0.00 | -0.04 | 0.00 | 0.00 | 0.00 | 0.00 | 0.04 | 0.00 | 0.17 | 0.00 | 0.41 | | 0.31 | 0.17 | 0.00 | 0.02 | 0.00 |
| F15 | 0.00 | -0.02 | 0.00 | 0.02 | 0.00 | 0.00 | 0.00 | 0.00 | 0.06 | 0.00 | 0.00 | 0.00 | 0.01 | 0.02 | 0.08 | 0.09 | 0.31 | | 0.24 | 0.07 | 0.09 | 0.00 |
| F16 | 0.02 | 0.00 | 0.00 | 0.00 | 0.00 | 0.00 | 0.00 | 0.00 | 0.00 | 0.00 | 0.00 | 0.02 | 0.05 | 0.03 | 0.00 | 0.05 | 0.17 | 0.24 | | 0.08 | 0.16 | 0.13 |
| F17 | 0.00 | 0.02 | 0.00 | 0.02 | 0.00 | 0.00 | 0.07 | 0.00 | 0.00 | 0.03 | 0.02 | 0.00 | 0.06 | 0.00 | 0.05 | 0.00 | 0.00 | 0.07 | 0.08 | | 0.23 | 0.25 |
| F18 | 0.00 | 0.04 | 0.00 | 0.01 | 0.00 | 0.00 | 0.00 | 0.00 | 0.03 | 0.01 | 0.00 | 0.00 | 0.00 | 0.03 | 0.03 | 0.07 | 0.02 | 0.09 | 0.16 | 0.23 | | 0.41 |
| F19 | 0.01 | 0.00 | 0.00 | 0.01 | 0.00 | 0.03 | 0.02 | 0.01 | 0.00 | 0.04 | 0.04 | 0.00 | 0.03 | -0.02 | 0.12 | 0.00 | 0.00 | 0.00 | 0.13 | 0.25 | 0.41 | |

**Table S3.** Raw Zero-Order Bivariate Correlation Matrix Among All Network Nodes in the Analytic Sample (N = 1,078)

| Variable | IC | CF | WM | F1 | F2 | F3 | F4 | F5 | F6 | F7 | F8 | F9 | F10 | F11 | F12 | F13 | F14 | F15 | F16 | F17 | F18 | F19 |
|---|---|---|---|---|---|---|---|---|---|---|---|---|---|---|---|---|---|---|---|---|---|---|
| **IC** | **1** | | | | | | | | | | | | | | | | | | | | | |
| **CF** | .711** | **1** | | | | | | | | | | | | | | | | | | | | |
| **WM** | .649** | .721** | **1** | | | | | | | | | | | | | | | | | | | |
| **F1** | .310** | .312** | .284** | **1** | | | | | | | | | | | | | | | | | | |
| **F2** | .250** | .239** | .213** | .722** | **1** | | | | | | | | | | | | | | | | | |
| **F3** | .323** | .332** | .302** | .498** | .424** | **1** | | | | | | | | | | | | | | | | |
| **F4** | .320** | .320** | .287** | .485** | .416** | .762** | **1** | | | | | | | | | | | | | | | |
| **F5** | .323** | .325** | .283** | .520** | .470** | .749** | .767** | **1** | | | | | | | | | | | | | | |
| **F6** | .280** | .291** | .243** | .584** | .602** | .610** | .636** | .727** | **1** | | | | | | | | | | | | | |
| **F7** | .328** | .326** | .298** | .526** | .496** | .645** | .657** | .706** | .704** | **1** | | | | | | | | | | | | |
| **F8** | .315** | .305** | .261** | .520** | .485** | .659** | .696** | .744** | .712** | .731** | **1** | | | | | | | | | | | |
| **F9** | .229** | .225** | .187** | .564** | .613** | .434** | .451** | .504** | .614** | .530** | .565** | **1** | | | | | | | | | | |
| **F10** | .292** | .285** | .251** | .564** | .558** | .537** | .555** | .584** | .639** | .596** | .641** | .803** | **1** | | | | | | | | | |
| **F11** | .278** | .271** | .249** | .565** | .597** | .449** | .440** | .509** | .592** | .517** | .527** | .747** | .719** | **1** | | | | | | | | |
| **F12** | .316** | .326** | .283** | .517** | .462** | .626** | .618** | .671** | .611** | .621** | .668** | .587** | .684** | .631** | **1** | | | | | | | |
| **F13** | .272** | .273** | .253** | .521** | .530** | .493** | .473** | .560** | .595** | .541** | .568** | .654** | .674** | .775** | .683** | **1** | | | | | | |
| **F14** | .252** | .246** | .220** | .533** | .547** | .475** | .458** | .544** | .599** | .531** | .552** | .676** | .672** | .776** | .649** | .856** | **1** | | | | | |
| **F15** | .266** | .257** | .232** | .537** | .534** | .512** | .500** | .574** | .623** | .551** | .580** | .645** | .671** | .715** | .682** | .782** | .831** | **1** | | | | |
| **F16** | .313** | .309** | .274** | .530** | .525** | .516** | .518** | .567** | .606** | .563** | .583** | .650** | .684** | .704** | .669** | .755** | .792** | .805** | **1** | | | |
| **F17** | .324** | .335** | .289** | .530** | .500** | .567** | .587** | .602** | .606** | .600** | .625** | .593** | .663** | .604** | .679** | .646** | .663** | .708** | .734** | **1** | | |
| **F18** | .336** | .350** | .300** | .545** | .519** | .554** | .548** | .611** | .632** | .604** | .615** | .609** | .668** | .680** | .701** | .730** | .736** | .761** | .793** | .803** | **1** | |
| **F19** | .337** | .341** | .300** | .540** | .497** | .589** | .595** | .630** | .624** | .621** | .649** | .590** | .673** | .617** | .717** | .679** | .676** | .716** | .767** | .801** | .855** | **1** |
